# Ship Fever, so Called; Its History, Nature, and Best Treatment

**Published:** 1850-11

**Authors:** 


*[illegible]hip Fever, so called; its History, Nature, and best Treatment. The Fiske Fund Prize Dissertation for* 1849. By Henry Grafton Clark, M. D. Printed by order of the Rhode Island Medical Society. Boston: Ticknor, Reed & Fields. 8vo., pp. 48.

This, as the title page imports, is a publication of the Essay which gained the Fiske Fund Prize for 1849. The author, from his position as Port Physician of the city of Boston, has enjoyed ample opportunity for studying the disease of which the essay treats, having had, as he states, personal familiarity with nearly two thousand cases. Cases of Ship Fever, so called, in so far as the observations of Dr. Clark have extended, are cases of *Typhus* as distinguished from *Typhoid.* He has found no difficulty in practically making the distinction between these two forms of fever, adopting as the basis of discrimination, the diagnostic points laid down in the Treatise on Fever by Dr. Bartlett.

Dr. Clark is of opinion that Typhus "is very clearly different from any fever which has hitherto been known in this country"—an opinion, probably, much too broadly and positively expressed. He regards it as "an entirely distinct disease" from the Typhoid fevers of France and New England. The same criticism, in our opinion, is applicable to the latter opinion. The conclusion adopted in view of the autopsical examinations made by the author is, that "Peyers glands are usually unaffected in any form of ship fever." The records of the appearances of the intestine in three cases are given as illustrations. It would have been more satisfactory if the observations with reference to this point, in a larger number of ases, had been submitted; or even if the author had stated the number dissections upon which the above conclusion is predicated.

Two-thirds of the mortality of the cases falling under th 's observation have been owing to secondary affections, which may be classed nder two forms, viz.:

1. General dropsy, which is often accompanied by swelling and sloughing, or suppuration of the parotid and other glands, and occasionally by suffocative œdema of the glottis.

2. A diarrhœa, or dysentery, which is usually dependent upon inflammation of the ileum and colon.

The number of instances in which the affection became developed among those brought into contact with the sick, sufficed to show the contagiousness of the disease. Facts also were observed which appeared to establish that the disease is infectious as well as contagious. With proper attention to cleanliness and ventilation, however, its communicability, in either mode, may be restricted within very narrow limits.

The remarks on Treatment are judicious, but contain nothing new.

We have thus given a condensed summary of the several points which the investigations of Dr. C. tend to establish. We have read the essay with interest, but we could not forbear wishing that, in connection with conclusions, more of the data had been furnished.

